# Structure of epidemic models: toward further applications in economics

**DOI:** 10.1007/s42973-021-00094-8

**Published:** 2021-08-31

**Authors:** Toshikazu Kuniya

**Affiliations:** 1-1 Rokkodai-cho, Nada-ku, Kobe, 657-8501 Japan

**Keywords:** Epidemic model, Reproduction number, Intervention, Behavior change, C62, I10

## Abstract

In this paper, we review the structure of various epidemic models in mathematical epidemiology for the future applications in economics. The heterogeneity of population and the generalization of nonlinear terms play important roles in making more elaborate and realistic models. The basic, effective, control and type reproduction numbers have been used to estimate the intensity of epidemic, to evaluate the effectiveness of interventions and to design appropriate interventions. The advanced epidemic models includes the age structure, seasonality, spatial diffusion, mutation and reinfection, and the theory of reproduction numbers has been generalized to them. In particular, the existence of sustained periodic solutions has attracted much interest because they can explain the recurrent waves of epidemic. Although the theory of epidemic models has been developed in decades and the development has been accelerated through COVID-19, it is still difficult to completely answer the uncertainty problem of epidemic models. We would have to mind that there is no single model that can solve all questions and build a scientific attitude to comprehensively understand the results obtained by various researchers from different backgrounds.

## Introduction

The first study of a mathematical epidemic model was conducted by Bernoulli (1760) in order to discuss the effectiveness of the universal inoculation against smallpox. One of the most celebrated epidemic models is the susceptible–infective–removed (SIR) model, which was developed by Kermack and McKendrick (1927) to simulate the epidemic dynamics in a closed population. Against the coronavirus disease 2019 (COVID-19) pandemic, many epidemic models have been constructed to predict the epidemic curve and evaluate the effectiveness of interventions (Abdullah et al., 2021; Acuña-Zegarra et al., 2020; Bhadauria et al., 2021; Buhat et al., 2021; Kim et al., 2020; Kuniya & Inaba, 2020; Liu et al., 2020; Mandal et al., 2020; Wang, 2020; Zeb et al., 2020; Zhang et al., 2021). Most of these models are related to the original SIR model.

Through COVID-19, epidemic models have attracted much attention from researchers in many fields, not limited to mathematical epidemiology. In particular, as COVID-19 has given huge impacts on the global economy, many economists have become interested in the application of epidemic models to the economic considerations (Avery et al., 2020). Before COVID-19, the possibility of the cross-discipline collaboration between economics and mathematical epidemiology was explored by Klein et al. (2007). They raised the following criticisms toward typical epidemic models from the viewpoint of economists:

- *Most models regard that hosts in epidemics are freely mixing and the contact rate is incapable of change.*
- *Few models address the problem of rational behavior at the individual level.*
- *Behavior of agents in the context of externalities is often ignored.*

Klein et al. (2007) insisted that behavioral choices should be incorporated into epidemic models to improve the accuracy of estimations and develop appropriate policies. Similar issues were addressed by Philipson (2000) from the viewpoint of economic epidemiology. However, to our knowledge, the majority of epidemic models has disregarded these issues even in the time of COVID-19. The purpose of this paper is to review the previous studies on epidemic models in mathematical epidemiology and to indicate possible directions to improve epidemic models for further applications in economics.

In mathematical epidemiology, some epidemic models have taken into account the behavior change of individuals by introducing the additional nonlinearity into incidence rates. For instance, Capasso and Serio (1978) generalized the incidence rate in the SIR model to the saturated or non-monotone one to capture the situation where individuals reduce the opportunity of contacts when the number of infective individuals becomes large. As such saturation or psychological effects would be a key idea to answer the aforementioned problems, we will review it and related studies in this paper. Moreover, as the heterogeneity of population would also be a key idea to consider the individual’s rational behavior, we will review the previous studies on structured epidemic models including multi-group models and age-structured models. We will also review the concepts of reproduction numbers (basic, effective, type and control reproduction numbers) because they play important roles in evaluating the effectiveness and impact of intervention policies.

The organization of this paper is as follows. In Sect. 2, we introduce the basic epidemic models (without intervention) including SIR, SEIR, SIS and SIRS models. We review how to incorporate the multi-group structure into them, and how to define the basic and effective reproduction numbers for them. In Sect. 3, we review how to take into account the effects of intervention policies in epidemic models. We also review the concepts of control and type reproduction numbers that play important roles in determining the target values of intervention policies to curb the epidemic. In Sect. 4, we review the previous results on the people’s behavior changes in epidemic models. We review some types of nonlinear incidence rates and show a simulation result in which the time delay and the sensitivity of the behavior change play essential roles in the occurrence of the recurrent epidemic waves. In Sect. 5, we review advanced epidemic models in the forms of PDEs or non-autonomous systems. They includes the age structure, seasonality, spatial diffusion, mutation and reinfection, and the theory of reproduction numbers can be generalized to them. Finally, Sect. 6 is devoted to the discussion.

## Basic models and concepts

### Basic models

**Fig. 1:**
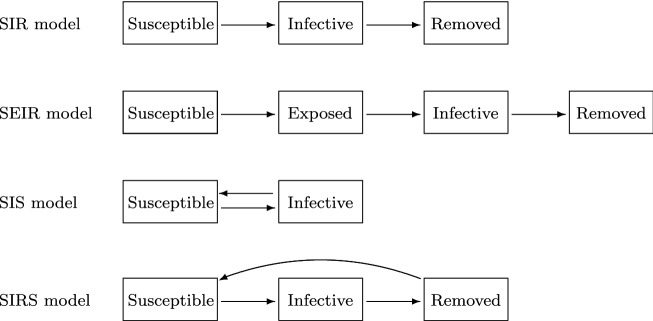
Transfer diagram of SIR, SEIR, SIS and SIRS models

In the SIR model, the total population is divided into three classes called susceptible, infective (or infected) and removed (or recovered). Individuals in the susceptible class can transfer to the infective class by infection, and individuals in the infective class can transfer to the removed class by recovery or quarantine (see the first row in Fig. 1). The SIR model without vital dynamics (births and deaths) is formulated by the following system of ordinary differential equations (Kermack & McKendrick, 1927, Section 3.2):

1 $$\begin{aligned} \left\{ \begin{array}{l} \displaystyle S'(t) = - \lambda (t)S(t), \\ \displaystyle I'(t) = \lambda (t)S(t) - \gamma I(t), \\ \displaystyle R'(t) = \gamma I(t), \end{array} \right. \end{aligned}$$

where *S*(*t*), *I*(*t*) and *R*(*t*) denote the susceptible, infective and removed populations at time *t*, respectively. $\gamma$ denotes the removal rate such that $1/\gamma$ means the average period of infectiousness. $\lambda (t)$ is called the force of infection at time *t*, which typical forms are

2 $$\begin{aligned} \lambda = \beta I \ \ (\mathrm {mass \ action \ incidence}) \ \ \mathrm {and} \ \ \lambda = \frac{\beta I}{N} \ \ (\mathrm {standard \ incidence}), \end{aligned}$$

where $\beta$ denotes the disease transmission coefficient and $N = S+I+R$ denotes the total population. The mass action incidence has been widely used but it disregards the saturation effect of the number of contacts. The standard incidence reflects the saturation effect of the number of contacts in a sufficiently large-scale population and has been usually adopted for modeling sexually transmitted diseases (Inaba, 2017, Section 5.1.1). If the total population *N* is constant, then there is no essential difference between both of these two incidence rates. In this paper, unless otherwise noted, each parameter is assumed to be positive.

Usually, epidemic models are constructed by adding (resp. removing) class(es) to (resp. from) the original SIR model. For instance, the susceptible–exposed–infective–removed (SEIR) model is constructed by adding the exposed class to the SIR model (see the second row in Fig. 1). The system (1) can then be reformulated as follows:

$$\begin{aligned} \left\{ \begin{array}{l} \displaystyle S'(t) = - \lambda (t)S(t), \\ \displaystyle E'(t) = \lambda (t) S(t) - \varepsilon E(t), \\ \displaystyle I'(t) = \varepsilon E(t) - \gamma I(t), \\ \displaystyle R'(t) = \gamma I(t), \end{array} \right. \end{aligned}$$

where *E*(*t*) denotes the exposed population at time *t* and $\varepsilon$ is the transition rate from *E* to *I*. If we regard *E* as the latent class, then the force of infection $\lambda$ is given as similar to (2). On the other hand, if we regard *E* as the asymptomatic infective class, then the typical forms of $\lambda$ are

$$\begin{aligned} \lambda = \beta _1 E + \beta _2 I \ \ (\mathrm {mass \ action}) \ \ \mathrm {and} \ \ \lambda = \frac{\beta _1 E + \beta _2 I}{N} \ \ (\mathrm {standard}), \end{aligned}$$

where $\beta _1$ and $\beta _2$ are the disease transmission coefficients for the asymptomatic and symptomatic infections, respectively. In this case, $N=S+E+I+R$. In COVID-19, the asymptomatic infection has been regarded as an important transmission path (He et al., 2020) and the asymptomatic class has been incorporated into models (Kuniya & Inaba, 2020; Zhang et al., 2021).

How long the immunity to infection will last is one of the most attracting topics in COVID-19 (Dan et al., 2021). If the immunity is not permanent in an epidemic model, then there would exist a transfer path back to the susceptible class. SIS and SIRS models are typical examples of such models (see the third and fourth rows in Fig. 1). The SIS and SIRS models without vital dynamics are given by

$$\begin{aligned} \left\{ \begin{array}{l} \displaystyle S'(t) = - \lambda (t)S(t) + \gamma I(t), \\ \displaystyle I'(t) = \lambda (t)S(t) - \gamma I(t), \\ \end{array} \right. \end{aligned}$$

and

$$\begin{aligned} \left\{ \begin{array}{l} \displaystyle S'(t) = - \lambda (t)S(t)+\delta R(t), \\ \displaystyle I'(t) = \lambda (t)S(t) - \gamma I(t), \\ \displaystyle R'(t) = \gamma I(t)-\delta R(t), \end{array} \right. \end{aligned}$$

respectively, where $\delta$ denotes the transition rate from *R* to *S* and $\lambda$ is given similar as in (2). SIRS-type reinfection models have also been applied to COVID-19 (Good & Hawkes, 2020; Kassa et al., 2020).

We now briefly review some other epidemic and related models. SIRI-type models have been studied for diseases with relapse (van den Driessche & Zou, 2007), drug diseases (White & Comiskey, 2007) and fictional zombie diseases (Munz et al., 2009). MSIR-type models have been studied to consider the class *M* with passive immunity at birth (Hethcote, 2000). The idea of compartmental models has also been applied to model the viral infection of cells (Kitagawa et al., 2019; Nowak & Bangham, 1996), the spread of computer virus (Kephart & White, 1993; Muroya & Kuniya, 2015) and the spread of rumor (Kawachi, 2008). They are not essentially the same as the SIR model but consider specific infective agents such as virus and rumor spreader.

On the other hand, if we consider the vital dynamics (births and deaths), then the SIR model (1) can be reformulated as follows (Hethcote, 1976):

3 $$\begin{aligned} \left\{ \begin{array}{l} \displaystyle S'(t) = b - \lambda (t)S(t) -\mu S(t), \\ \displaystyle I'(t) = \lambda (t)S(t) - \left( \mu + \gamma \right) I(t), \\ \displaystyle R'(t) = \gamma I(t) - \mu R(t), \end{array} \right. \end{aligned}$$

where *b* and $\mu$ denote the birth and mortality rates, respectively. To distinguish the models without and with vital dynamics, the latter is sometimes called the endemic model (Hethcote, 2000). The justification of the constant birth rate *b* in model (3) is as follows: let *kN*(*t*) be the population of newborns at time *t*. In this case, the first equation in (3) is replaced by

4 $$\begin{aligned} S'(t) = kN(t) - \lambda (t)S(t) -\mu S(t). \end{aligned}$$

By adding the three equations of *S*, *I* and *R*, we obtain

$$\begin{aligned} N'(t) = (k - \mu ) N(t). \end{aligned}$$

This is the Malthus model with exact solution $N(t)=N(0)\mathrm{e}^{(k-\mu )t}$. Under the assumption that the nontrivial demographic steady state exists, $k=\mu$ and thus, *N*(*t*) is constant. We can then regard $b=kN=\mu N$ as a constant, and (3) is obtained.

### Multi-group models

**Fig. 2:**
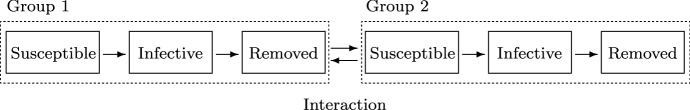
Conceptual diagram of the two-group SIR model

One common way to improve epidemic models is to incorporate the multi-group structure. In multi-group models, the heterogeneity (e.g., age, position, sex, etc.) of each individual can be indexed by a subscript. For instance, the SIR model (1) without vital dynamics can be reformulated into the following two-group model (see also Fig. 2):

5 $$\begin{aligned} \left\{ \begin{array}{ll} \displaystyle S_1'(t) = - \lambda _1(t)S_1(t), &{} \displaystyle S_2'(t) = - \lambda _2(t)S_2(t), \\ \displaystyle I_1'(t) = \lambda _1(t)S_1(t) - \gamma _1 I_1(t), &{} \displaystyle I_2'(t) = \lambda _2(t)S_2(t) - \gamma _2 I_2(t), \\ \displaystyle R_1'(t) = \gamma _1 I_1(t), &{}\displaystyle R_2'(t) = \gamma _2 I_2(t), \end{array} \right. \end{aligned}$$

where each symbol is similar to that in (1) but the subscript represents the group. For example, if we let subscripts 1 and 2 denote the male and female groups, respectively, then (5) can be a model for sexually transmitted diseases (Lajmanovich & Yorke, 1976). The interaction between different groups is considered in the forces of infection $\lambda _1,\lambda _2$, which typical forms are

$$\begin{aligned} \left\{ \ \begin{array}{lll} \displaystyle \lambda _1 = \beta _{11}I_1 + \beta _{12}I_2, &{} \lambda _2 = \beta _{21}I_1 + \beta _{22}I_2 &{} (\mathrm {mass \ action}) \ \ \mathrm {and} \\ \displaystyle \lambda _1 = \frac{\beta _{11}I_1}{N_1} + \frac{\beta _{12}I_2}{N_2}, \ &{} \displaystyle \lambda _2 = \frac{\beta _{21}I_1}{N_1} + \frac{\beta _{22}I_2}{N_2} \ &{} (\mathrm {standard}), \end{array} \right. \end{aligned}$$

where $\beta _{jk}$ denotes the disease transmission coefficient for infective individuals in group *k* to susceptible individuals in group *j*, and $N_j = S_j+I_j+R_j$ is the total population in group *j*. If we assume that two groups represent human and vector groups, then (5) can also be a model for vector-borne diseases (Bacaër, 2011). Other types of epidemic models with two-group structure have been also applied to COVID-19 (Acuña-Zegarra et al., 2020; Buhat et al., 2021).

In general, we can consider arbitrary *n* groups in the multi-group model. The two-group SIR model (5) can be generalized to the following *n*-group model:

6 $$\begin{aligned} \left\{ \begin{array}{l} \displaystyle S_j'(t) = - \lambda _j(t)S_j(t), \\ \displaystyle I_j'(t) = \lambda _j(t)S_j(t) - \gamma _j I_j(t), \\ \displaystyle R_j'(t) = \gamma _j I_j(t), \end{array} \right. \quad j = 1,2,\ldots , n, \end{aligned}$$

where the typical forms of force of infection $\lambda _j$ are

$$\begin{aligned} \left\{ \ \begin{array}{ll} \displaystyle \lambda _j = \sum _{k=1}^n \beta _{jk} I_k &{} (\mathrm {mass \ action}) \ \ \mathrm {and} \\ \displaystyle \lambda _j = \sum _{k=1}^n \frac{\beta _{jk}I_k}{N_k} \ &{} (\mathrm {standard}), \end{array} \quad j = 1,2,\ldots , n. \right. \end{aligned}$$

Multi-group epidemic models with $n \ (\ge 2)$ groups have been applied to sexually transmitted diseases to consider the activity of each individual (Murray, 2002, Section 10.4). Here, the activity implies the frequency of sexual contacts, and the population is divided into *n* subgroups according to the gender and the activity. On the other hand, if we consider the movement of individuals among different groups, then the *n*-group SIR model (6) can be modified into the following form:

$$\begin{aligned} \left\{ \begin{array}{l} \displaystyle S_j'(t) = - \lambda _j(t)S_j(t) - m_j S_j(t) + \sum _{k\ne j}m_{jk} S_k(t), \\ \displaystyle I_j'(t) = \lambda _j(t)S_j(t) - \gamma _j I_j(t)-m_j I_j(t) + \sum _{k\ne j} m_{jk} I_k(t), \\ \displaystyle R_j'(t) = \gamma _j I_j(t) - m_j R_j(t) + \sum _{k\ne j}m_{jk}R_k(t), \end{array} \right. \quad j = 1,2,\ldots , n, \end{aligned}$$

where $m_{jk}$ denotes the rate of movement from group *k* to group *j* and $m_j = \sum _{k\ne j} m_{kj}$. This type of model is often called a metapopulation model (Arino, 2009) or a model in patchy environment (Wang & Zhao, 2004). Other models related to the multi-group models are, for instance, multi-strain models (Otani et al., 2017) and network models (Kiss et al., 2017).

### Basic reproduction number

The basic reproduction number ${{\mathcal {R}}}_0$ is defined by the expected number of secondary cases produced by a typical infective individual in a completely susceptible population (Diekmann et al., 1990). Intuitively, ${{\mathcal {R}}}_0$ implies the strength of the epidemic and if ${{\mathcal {R}}}_0 > 1$, then an outbreak will occur, whereas if ${{\mathcal {R}}}_0 < 1$, then there will be no outbreak. The effective reproduction number ${{\mathcal {R}}}_t$ is defined by the expected number of secondary cases produced by a typical infective individual at calendar time *t* (Nishiura & Chowell, 2009). ${{\mathcal {R}}}_0$ and ${{\mathcal {R}}}_t$ have attracted much attention in COVID-19 and have been estimated by many authors (Ahammed et al., 2021; Linka et al., 2020).

In the SIR model (1) without vital dynamics, the differential equation of *I* can be written as

$$\begin{aligned} I'(t) = \left\{ \ \begin{array}{ll} \displaystyle \left[ \beta S(t) - \gamma \right] I(t) &{} (\mathrm {mass \ action}), \\ \displaystyle \left[ \frac{\beta S(t)}{N} - \gamma \right] I(t) \ &{} (\mathrm {standard}). \end{array} \right. \end{aligned}$$

Hence, we can obtain the explicit formula of ${{\mathcal {R}}}_t$ as

$$\begin{aligned} {{\mathcal {R}}}_t = \frac{\beta S(t)}{\gamma } \ \ (\mathrm {mass \ action}), \quad {{\mathcal {R}}}_t = \frac{\beta S(t)}{\gamma N} \ \ (\mathrm {standard}), \end{aligned}$$

so that if ${{\mathcal {R}}}_t > 1$, then $I'(t) > 0$ and the infective population will increase, whereas if ${{\mathcal {R}}}_t < 1$, then $I'(t)<0$ and the infective population will decrease. In a completely susceptible population, we have $S=N$, and hence, the explicit formula of ${{\mathcal {R}}}_0$ is given by

$$\begin{aligned} {{\mathcal {R}}}_0 = \frac{\beta N}{\gamma } \ \ (\mathrm {mass \ action}), \quad {{\mathcal {R}}}_0 = \frac{\beta }{\gamma } \ \ (\mathrm {standard}). \end{aligned}$$

**Fig. 3:**
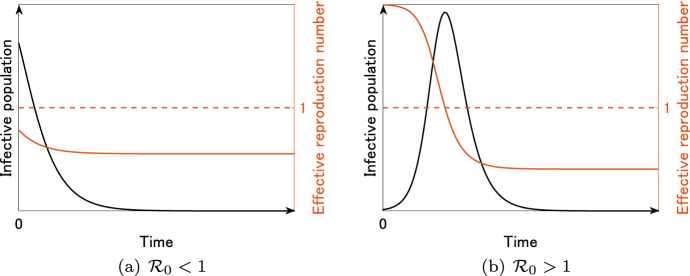
Time variation of the infective population *I*(*t*) (black) and the effective reproduction number ${{\mathcal {R}}}_t$ (red) for the SIR model (1) without vital dynamics

Figure 3 illustrates the typical epidemic curves generated by the SIR model (1) without vital dynamics. For ${{\mathcal {R}}}_0 < 1$, the infective population *I*(*t*) is monotone decreasing and there is no outbreak (Fig. 3a). For ${{\mathcal {R}}}_0 > 1$, the infective population *I*(*t*) is not monotone and an outbreak occurs (Fig. 3b). In both cases, ${{\mathcal {R}}}_t$ is monotone decreasing. However, in the case of ${{\mathcal {R}}}_0 > 1$, ${{\mathcal {R}}}_t$ crosses 1 at which the epidemic curve attains the peak (Fig. 3b).

On the other hand, for the SIR model (3) with vital dynamics, the explicit formula of the effective reproduction number ${{\mathcal {R}}}_t$ is given by

$$\begin{aligned} {{\mathcal {R}}}_t = \frac{\beta S(t)}{\mu +\gamma } \ \ (\mathrm {mass \ action}), \quad {{\mathcal {R}}}_t = \frac{\beta S(t)}{(\mu +\gamma ) N} \ \ (\mathrm {standard}). \end{aligned}$$

In a completely susceptible population, we have $S=N=b/\mu$, and hence, the explicit formula of the basic reproduction number ${{\mathcal {R}}}_0$ is given by

$$\begin{aligned} {{\mathcal {R}}}_0 = \frac{\beta N}{\mu +\gamma }=\frac{\beta b}{(\mu +\gamma ) \mu } \ \ (\mathrm {mass \ action}), \quad {{\mathcal {R}}}_0 = \frac{\beta }{\mu +\gamma } \ \ (\mathrm {standard}). \end{aligned}$$

**Fig. 4:**
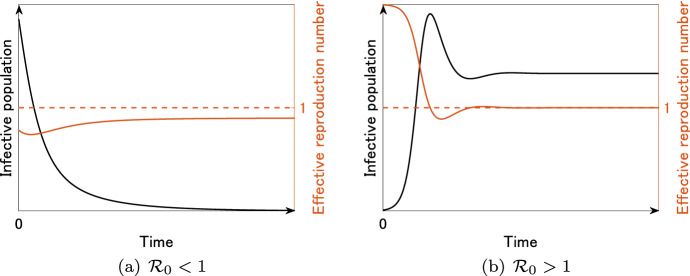
Time variation of the infective population *I*(*t*) (black) and the effective reproduction number ${{\mathcal {R}}}_t$ (red) for the SIR model (3) with vital dynamics

Figure 4 illustrates the typical epidemic curves generated by the SIR model (3) with vital dynamics. Similar to the case in Fig. 3a, if ${{\mathcal {R}}}_0 < 1$, then the infective population *I*(*t*) is monotone decreasing and there is no outbreak (Fig. 4a). However, note that ${{\mathcal {R}}}_t$ is not monotone in Fig. 4. For ${{\mathcal {R}}}_0 > 1$, the infective population *I*(*t*) converges to a positive steady state $I^*$ at which ${{\mathcal {R}}}_t = 1$ (Fig. 4b). Such $I^*$ is explicitly given by

$$\begin{aligned} I^* = \frac{b}{\mu +\gamma } \left( 1-\frac{1}{{{\mathcal {R}}}_0} \right) , \end{aligned}$$

which is positive if and only if ${{\mathcal {R}}}_0 > 1$. That is, in the SIR model (3) with vital dynamics, ${{\mathcal {R}}}_0$ is the threshold for the existence of the positive steady state, which is traditionally called the endemic equilibrium (Hethcote, 2000). In fact, ${{\mathcal {R}}}_0$ for model (3) satisfies the following threshold theorem:

**Threshold theorem** If ${{\mathcal {R}}}_0 \le 1$, then the disease-free equilibrium is globally asymptotically stable, whereas if ${{\mathcal {R}}}_0 > 1$, then the endemic equilibrium is globally asymptotically stable.

Here, the disease-free equilibrium is defined by the steady state at which there is no infective population (that is, $I=0$). Roughly speaking, the global asymptotic stability of an equilibrium means that every solution in a specific set converges to the equilibrium as time goes to infinity. Therefore, the above threshold theorem implies that either the disease-free or endemic equilibrium will eventually be attained depending on ${{\mathcal {R}}}_0$. This theorem suggests that ${{\mathcal {R}}}_0$ is an important threshold value for predicting the eventual dynamics of epidemic spreading, however, it excludes the possibility of periodic solutions that may explain the recurrent epidemic waves.

Mathematically, ${{\mathcal {R}}}_0$ is defined by the spectral radius of an operator called the next generation operator (Diekmann et al., 1990). For multi-group epidemic models (see Sect. 2.2), in many applications, ${{\mathcal {R}}}_0$ can be computed as the maximum eigenvalue of a matrix called the next generation matrix (van den Driessche & Watmough, 2002). Usually, the next generation matrix is written as ${\mathbf {K}}=(k_{ij})$, where $k_{ij}$ implies the expected number of secondary cases in group *i* produced by a typical infective individual in group *j* when the population is completely susceptible. For instance, the next generation matrix for the two-group SIR model (5) is given by

$$\begin{aligned} \left( \begin{array}{cc} \displaystyle \frac{\beta _{11}N_1}{\gamma _1} &{} \displaystyle \frac{\beta _{12}N_1}{\gamma _2} \\ \displaystyle \frac{\beta _{21}N_2}{\gamma _1} &{} \displaystyle \frac{\beta _{22}N_2}{\gamma _2} \end{array} \right) \ (\mathrm {mass \ action}), \quad \left( \begin{array}{cc} \displaystyle \frac{\beta _{11}}{\gamma _1} &{} \displaystyle \frac{\beta _{12}}{\gamma _2}\frac{N_1}{N_2} \\ \displaystyle \frac{\beta _{21}}{\gamma _1}\frac{N_2}{N_1} &{} \displaystyle \frac{\beta _{22}}{\gamma _2} \end{array} \right) \ (\mathrm {standard}). \end{aligned}$$

${{\mathcal {R}}}_0$ is the spectral radius of such a next generation matrix. Threshold theorem of ${{\mathcal {R}}}_0$ holds not only for a two-group SIR model with vital dynamics but also for a general *n*-group SIR model with vital dynamics (Guo et al., 2006).

## Intervention

To evaluate the (positive or negative) effects of intervention would be one of the central purposes of mathematical modeling in both of economics and epidemiology. One of the key concepts is the control reproduction number ${{\mathcal {R}}}_\mathrm{c}$, which is the reproduction number when intervention is in place (Gumel et al., 2004).

### Modification of parameters

The simplest way to consider the effects of intervention is to modify some model parameter(s). For example, we may assume that the disease transmission coefficient $\beta$ in the SIR model (1) without vital dynamics is reduced to $(1-r) \beta$, $0<r<1$ by virtue of intervention such as social distancing. In such a case, the control reproduction number is given by ${{\mathcal {R}}}_\mathrm{c} = (1-r) {{\mathcal {R}}}_0$, and thus, *r* should be greater than the critical value $r^* := 1-1/{{\mathcal {R}}}_0$ to achieve ${{\mathcal {R}}}_\mathrm{c} < 1$. On the other hand, if we assume that the removal rate $\gamma$ in model (1) is raised to $\ell \gamma$, $\ell > 1$ by intervention such as isolation, then ${{\mathcal {R}}}_\mathrm{c} = {{\mathcal {R}}}_0/\ell$, and thus, $\ell$ should be greater than the critical value $\ell ^* := {{\mathcal {R}}}_0$ to achieve ${{\mathcal {R}}}_\mathrm{c} < 1$.

### Addition of treatment classes

**Fig. 5:**
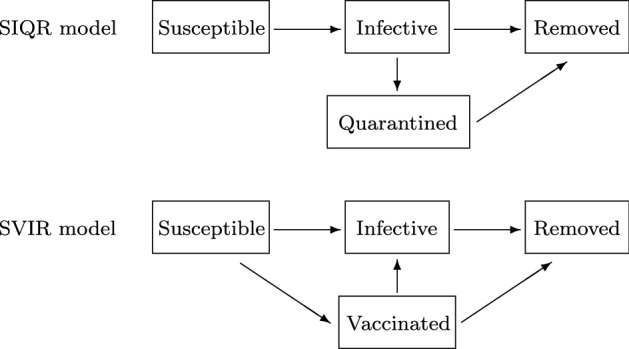
Transfer diagram of SIQR and SVIR models

The other common way to study the effects of intervention is to add new treatment classes to epidemic models. For instance, quarantined population is often denoted by *Q*, and epidemic models with class *Q* have been studied for decades (Hoppensteadt, 1974; Feng & Thieme, 1995). Traditionally, the quarantine of infective individuals has been studied by SIQR-type models. A typical SIQR model without vital dynamics is formulated as follows (see also Fig. 5).

7 $$\begin{aligned} \left\{ \begin{array}{l} \displaystyle S'(t) = - \lambda (t)S(t), \\ \displaystyle I'(t) = \lambda (t) S(t) - ( \gamma + q) I(t), \\ \displaystyle Q'(t) = q I(t) - \eta Q(t), \\ \displaystyle R'(t) = \gamma I(t) + \eta Q(t), \end{array} \right. \end{aligned}$$

where *q* denotes the quarantine rate and $\eta$ denotes the transition rate from *Q* to *R*. The meaning of the other symbols is similar to those in the SIR model (1). SIQR-type models have been applied to COVID-19 (Abdullah et al., 2021; Bhadauria et al., 2021; Mandal et al., 2020; Zeb et al., 2020). On the other hand, SQIR-type models have also been studied to consider the quarantine of susceptible individuals (Safi & Gumel, 2013; Algehyne & Din, 2021). SQIR-type models seem to correspond to the SVIR-type models, where *V* denotes the vaccinated population (Kribs-Zaleta & Velasco-Hernández, 2000; Liu et al., 2008). A typical SVIR model without vital dynamics is formulated as follows (see also Fig. 5).

8 $$\begin{aligned} \left\{ \begin{array}{l} \displaystyle S'(t) = - [ \lambda (t) + v ] S(t), \\ \displaystyle V'(t) = vS(t)- [ \sigma \lambda (t) + \zeta ] V(t), \\ \displaystyle I'(t) = \lambda (t) [ S(t)+\sigma V(t) ] - \gamma I(t), \\ \displaystyle R'(t) = \gamma I(t) + \zeta V(t), \end{array} \right. \end{aligned}$$

where *v* denotes the vaccination rate, $\sigma < 1$ denotes the reduction coefficient to the disease transmission coefficient ($1-\sigma$ is the vaccine efficacy) and $\zeta$ denotes the transition rate from *V* to *R*.

The control reproduction number ${{\mathcal {R}}}_\mathrm{c}$ for the SIQR model (7) is given by

$$\begin{aligned} {{\mathcal {R}}}_\mathrm{c} = \frac{\gamma }{\gamma + q} {{\mathcal {R}}}_0, \end{aligned}$$

where ${{\mathcal {R}}}_0$ is the basic reproduction number for the SIR model (1) without vital dynamics. Thus, the quarantine rate *q* should be greater than the critical value $q^* := \gamma ({{\mathcal {R}}}_0-1)$ to achieve ${{\mathcal {R}}}_\mathrm{c} < 1$. On the other hand, the control reproduction number ${{\mathcal {R}}}_\mathrm{c}$ for the SVIR model (8) without vital dynamics is given by ${{\mathcal {R}}}_\mathrm{c}={{\mathcal {R}}}_0$ because there is no vaccinated individual in the completely susceptible population. However, for the following SVIR model with vital dynamics

$$\begin{aligned} \left\{ \begin{array}{l} \displaystyle S'(t) = b- [ \lambda (t) +\mu + v] S(t), \\ \displaystyle V'(t) = vS(t)- [ \sigma \lambda (t) + \mu +\zeta ] V(t), \\ \displaystyle I'(t) = \lambda (t) [ S(t)+\sigma V(t) ] - (\mu + \gamma ) I(t), \\ \displaystyle R'(t) = \gamma I(t) +\zeta V(t) -\mu R(t), \end{array} \right. \end{aligned}$$

the control reproduction number ${{\mathcal {R}}}_\mathrm{c}$ is given by, for $\zeta =0$,

$$\begin{aligned} {{\mathcal {R}}}_\mathrm{c} = \frac{\mu +\sigma v}{\mu + v} {{\mathcal {R}}}_0. \end{aligned}$$

Thus, the vaccination rate *v* should be greater than the critical value $v^* := \mu ({{\mathcal {R}}}_0-1)/(1-\sigma {{\mathcal {R}}}_0)$ to achieve ${{\mathcal {R}}}_\mathrm{c} < 1$. This can be achieved only if $\sigma {{\mathcal {R}}}_0 < 1$.

### Type reproduction number

As stated above, the basic reproduction number ${{\mathcal {R}}}_0$ for multi-group models is defined by the spectral radius of the next generation matrix. If we use the control reproduction number ${{\mathcal {R}}}_\mathrm{c}$ based on such ${{\mathcal {R}}}_0$, then it would provide only a critical value that is uniform for all groups. Type reproduction number ${{\mathcal {T}}}$ was introduced by Roberts and Heesterbeek (2003) to obtain a critical value that focuses on a specific group. More precisely, in a general *n*-group epidemic model, the type reproduction number ${{\mathcal {T}}}$ for group 1 is given by

$$\begin{aligned} {{\mathcal {T}}} = {\mathbf {e}}^{\mathrm{T}} {\mathbf {K}} \left[ {\mathbf {I}} - ({\mathbf {I}} - {\mathbf {P}}) {\mathbf {K}} \right] ^{-1} {\mathbf {e}}, \end{aligned}$$

where ${\mathbf {e}}$ is a column vector whose first element is 1 and the others are 0, ${\mathbf {K}}=(k_{ij})$ is the next generation matrix, ${\mathbf {I}}$ is the $n \times n$ identity matrix, and ${\mathbf {P}}$ is the projection matrix whose (1, 1) element is 1 and the others are 0. If $n=2$, then we obtain

$$\begin{aligned} {{\mathcal {T}}} = k_{11} + \frac{k_{12}k_{21}}{1-k_{22}}. \end{aligned}$$

If $k_{22}>1$, then the infective population in group 2 can reproduce by itself, and thus, we can not control the disease by intervention restricted to group 1. If $k_{22} < 1$, then ${{\mathcal {T}}} > 1$ is equivalent to ${{\mathcal {R}}}_0 > 1$ (Roberts & Heesterbeek, 2003, Section 2). In this case, we may assume that intervention on group 1 reduces $k_{1j}$ to $(1-r) k_{1j}$, $j=1,2$, and thus, ${{\mathcal {T}}}$ is reduced to $(1-r){{\mathcal {T}}}$. The critical value $r^*$ to make the reproduction number equal to 1 can then be obtained as $r^* := 1-1/{{\mathcal {T}}}$. That is, $r>r^*$ is sufficient to curb the epidemic.

## Behavior change

### Nonlinear terms

One way to intrinsically consider the behavior change of people in epidemic models is to introduce new nonlinear terms. For instance, the force of infection term in the SIR model (1) or (3) can be generalized to $\lambda =g(I)$ satisfying the following assumptions:

1. $g(0)=0$ and $g(I)>0$ for all $I>0$;
2. *g* is differentiable on ${\mathbb {R}}_+$.

Capasso and Serio (1978) further considered the following assumptions to take into account the saturation or psychological effects:

(A3) There exists a constant $c > 0$ such that $g(I)\le c$ for all $I > 0$;
(A4) $g'$ is bounded on ${\mathbb {R}}_+$ and $g(I)\le g'(0) I$ for all $I > 0$.

Typical examples of such *g* are as follows (see also Table 1):

$$\begin{aligned} \begin{array}{ll} \displaystyle g(I) = \frac{\beta I}{1+\alpha I} &{} (\mathrm {saturation \ effect}), \\ \displaystyle g(I) = \frac{\beta I}{1+\alpha I^p}, \ \ p > 1 \ \ &{} (\mathrm {psychological \ effect}). \end{array} \end{aligned}$$

**Table 1:** Example of nonlinear incidence rates

*g*(*I*)	Description	References
$\beta I$	Mass action incidence	Kermack and McKendrick (1927)
$\displaystyle \frac{\beta I}{N}$	Standard incidence	Hethcote (2000)
$\displaystyle \frac{\beta I}{1+\alpha I}$	Saturation effect	Capasso and Serio (1978)
$\displaystyle \frac{\beta I}{1+\alpha I^p}, \ p > 1$	Psychological effect	Xiao and Ruan (2007)
$\beta I^p, \ p \ne 1$	General incidence	Liu et al. (1986)

**Fig. 6:**
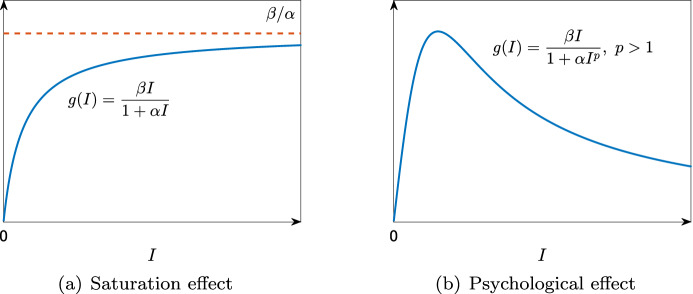
*g*(*I*) for considering **a** the saturation effect and **b** the psychological effect

The saturation effect implies that the force of infection will be saturated at a certain level when the infective population becomes large (Fig. 6a).

The psychological effect implies that the force of infection will decrease when the infective population becomes large (Fig. 6b). Both of these effects are based on the idea that people may tend to reduce the number of contacts when there are many infected individuals. Capasso and Serio (1978) stated that the idea of these effects were suggested after the study of the cholera epidemic spread in Bari in 1973. We can guess that these effects could be suitable for widely broadcasted and cautioned diseases such as COVID-19. In fact, some authors have considered the saturation effect in epidemic models applied to COVID-19 (Bhadauria et al., 2021). If the function *g* satisfies assumptions (A1)–(A4) and $g'>0$ and $g''\le 0$ on ${\mathbb {R}}_+$, then the basic reproduction number ${{\mathcal {R}}}_0$ for the SIR model (3) with $\lambda =g(I)$ satisfies the threshold theorem: if ${{\mathcal {R}}}_0 \le 1$, then the disease-free equilibrium is globally asymptotically stable, whereas if ${{\mathcal {R}}}_0 > 1$, then the endemic equilibrium is globally asymptotically stable (Korobeinikov, 2007). Thus, in this case, no periodic solution exists. Although $g(I)=\beta I^p$, $p\ne 1$ does not satisfy assumptions (A3) and (A4), it was shown by Liu et al. (1986) that a periodic solution can exist by the Hopf bifurcation.

The nonlinearity can be introduced into terms other than the force of infection. Perra et al. (2011) assumed in their model that $k_1 S(t) [1-\mathrm{e}^{-k_2 I(t)}]$ susceptible individuals change their behavior per unit time, where $k_1$ and $k_2$ are positive constants. This idea was applied to COVID-19 modeling by Kim et al. (2020).

### Time delay

Time delay is known as one of the key factors that causes periodic solutions in epidemic models (Hethcote & Levin, 1989). Cooke (1979) studied an epidemic model with force of infection $\lambda (t)=\beta I(t-\tau )$ with fixed time delay $\tau$ to consider the spread of a vector-borne disease. Beretta and Takeuchi (1995) studied an SIR model with a more general distributed time delay $\lambda (t) = \beta \int _0^\infty f(\sigma ) I(t-\sigma ) \mathrm{d}\sigma$, where *f* is a non-negative distribution on ${\mathbb {R}}_+$, that is, $\int _0^\infty f(\sigma ) \mathrm{d}\sigma = 1$. McCluskey (2010) proved the threshold theorem of the basic reproduction number ${{\mathcal {R}}}_0$ for the SIR model (3) with delayed forces of infection $\lambda (t) = \beta I(t-\tau )$ and $\lambda (t) = \beta \int _0^\tau f(\sigma ) I(t-\sigma )\mathrm{d}\sigma$: if ${{\mathcal {R}}}_0 \le 1$, then the disease-free equilibrium is globally asymptotically stable, whereas if ${{\mathcal {R}}}_0 > 1$, then the endemic equilibrium is globally asymptotically stable. Thus, in such case, there is no possibility of periodic solutions.

Motivated by the idea of the saturated incidence rate, we now consider the SIR model (3) with the following force of infection:

9 $$\begin{aligned} \lambda (t)=\frac{\beta I(t)}{1+\alpha \int _0^\infty f(\sigma ) I(t-\sigma ) \mathrm{d}\sigma }, \end{aligned}$$

where *f* is the truncated exponential distribution

10 $$\begin{aligned} f(\sigma )= \left\{ \begin{array}{ll} 0, &{} \sigma < \tau , \\ k\mathrm{e}^{-k(\sigma -\tau )}, \ &{} \sigma \ge \tau , \end{array} \right. \end{aligned}$$

**Fig. 7:**
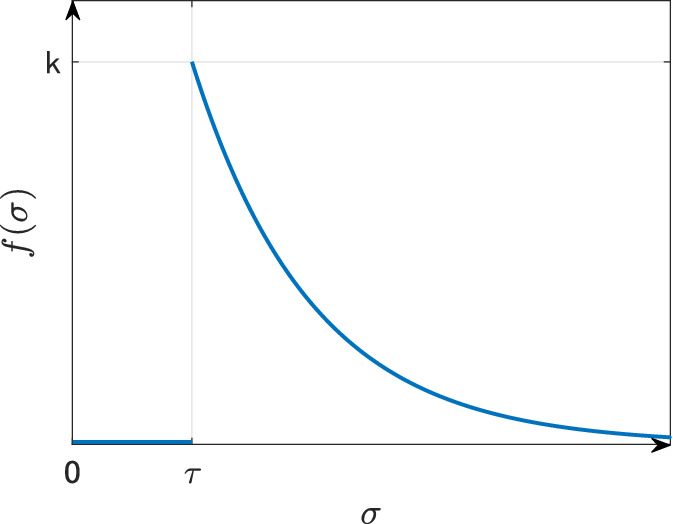
Truncated exponential distribution given by (10)

and *k* and $\tau$ are positive constants (see also Fig. 7). By regarding $\tau$ as a bifurcation parameter, we can numerically check that a periodic solution exists for some parameter sets.^1^ For instance, setting parameters

11 $$\begin{aligned} b = \mu = \frac{1}{100}, \quad \gamma = 1, \quad {{\mathcal {R}}}_0 = 2, \quad \alpha = 10, \quad \beta = \frac{{{\mathcal {R}}}_0 (\mu +\gamma ) \mu }{b}, \quad k= 1, \end{aligned}$$

we can numerically check that the endemic equilibrium is stable for $\tau = 14$ (Fig. 8a), whereas it is unstable and a periodic solution exists for $\tau =16$ (Fig. 8b). In this case, we can check that the destabilization of the endemic equilibrium occurs at $\tau = \tau _\mathrm{c} \approx 15$. Such critical value $\tau _\mathrm{c}$ can be calculated for each $\alpha$ and we can plot the parameter region where the periodic solution exists or not (Fig. 9). From Fig. 9, we can conjecture that the time delay $\tau$ and the sensitivity of the behavior change $\alpha$ play essential roles in the occurrence of the recurrent epidemic waves.

**Fig. 8:**
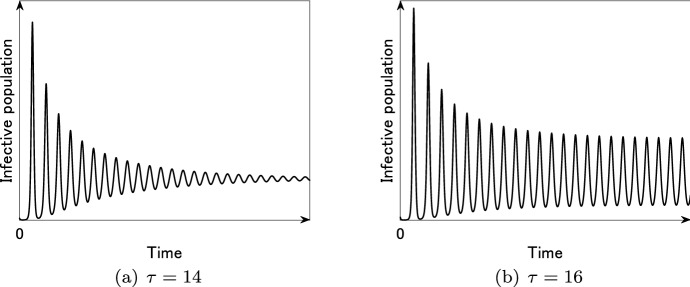
Time variation of the infective population in the SIR model (3) with the force of infection (9), the truncated exponential distribution (10) and parameters (11)

**Fig. 9:**
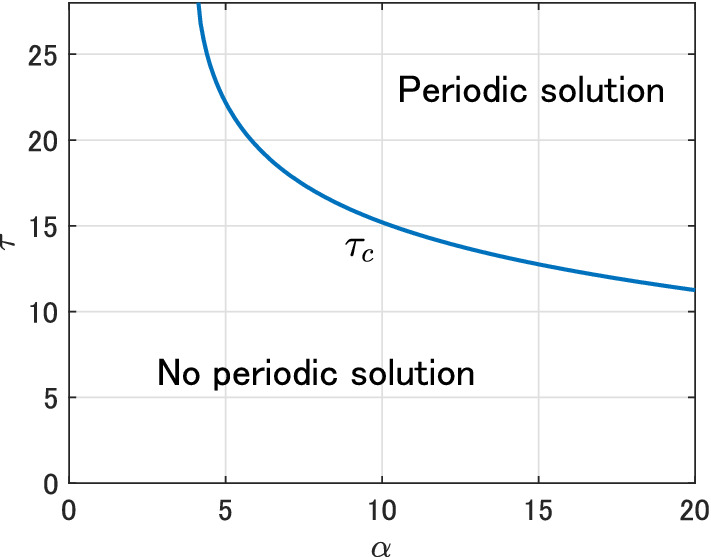
Parameter region where the periodic solution exists or not

**Fig. 10:**
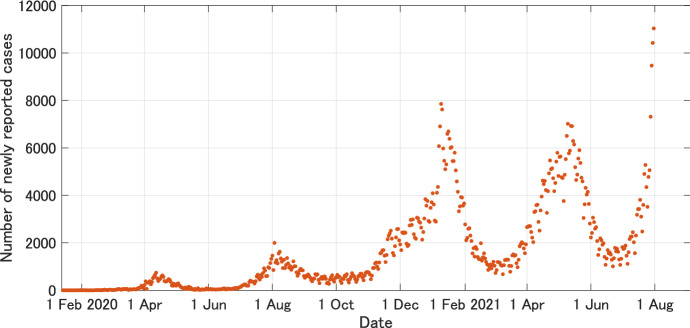
Daily number of newly reported cases of COVID-19 in Japan from 14 January, 2020 to 31 July, 2021 (WHO, 2021)

In the classical SIR model (1) without vital dynamics, the epidemic curve has at most one peak as shown in Fig. 3. However, in COVID-19, multiple peaks have been observed in many countries (see Fig. 10 for the case of Japan). In the early stage of COVID-19, the author used a one-peak model to predict the long-term behavior of the epidemic (Kuniya, 2020a). However, it was revealed that such a model is rarely to be close to the actual data if interventions are taken and people’s behavior changes in response to the epidemic (Kuniya, 2020b). One-peak models might have to be used for the purpose of assessing the short-term intensity of the epidemic, and it may be better to use models with behavior change to understand the long-term dynamics if the epidemic is frequently announced and people tend to respond to it.

### Switching

Switching is also an important concept to consider the people’s behavior change. Time-dependent parameters have often been considered in the applications to COVID-19 (Acuña-Zegarra et al., 2020; Liu et al., 2020). They enable us to consider the time variation of parameters in each event and seem to suitable for evaluating the effects of periodic/non-periodic interventions. We can regard that the solution obeys different dynamical systems in each time interval.

On the other hand, piecewise functions have also been considered in switched systems for COVID-19 (Wang, 2020). For instance, it is assumed that $\lambda = g(I)$ and

$$\begin{aligned} g(I) = \left\{ \begin{array}{ll} \beta _1 I, \quad &{} I \le I_\mathrm{c}, \\ \beta _2 I, &{} I > I_\mathrm{c}, \end{array} \right. \end{aligned}$$

for some $I_\mathrm{c} > 0$. We can regard such *g* is a discontinuous nonlinear incidence rate and such a system as a hybrid dynamical system. Hybrid dynamical systems can exhibit characteristic dynamics such as pseudoequilibria and sliding-modes (Wang et al., 2016).

## Further advanced models

### Age structure

Age structure is the key concept to capture the age-specific disease dynamics (Iannelli, 1995; Inaba, 2017). In the original epidemic model by Kermack and McKendrick (1927), the infection age (time elapsed since the infection) was considered. Let *I*(*t*, *a*) be the infective population of infection age *a* at time *t*. The SIR model (1) without vital dynamics can be generalized to the following coupled system of ordinary differential equations (ODEs) and a partial differential equation (PDE):

12 $$\begin{aligned} \left\{ \begin{array}{l} \displaystyle S'(t) = - \lambda (t) S(t), \\ \displaystyle \left( \frac{\partial }{\partial t} + \frac{\partial }{\partial a} \right) I(t,a) = - \gamma (a) I(t,a), \quad I(t,0) = \lambda (t) S(t), \\ \displaystyle R'(t) = \int _0^\infty \gamma (a) I(t,a) \mathrm{d}a, \end{array} \right. \end{aligned}$$

where $\gamma (a)$ denotes the removal rate at infection age *a*. The force of infection in the mass action law is given by

$$\begin{aligned} \lambda (t) = \int _0^\infty \beta (a) I(t,a) \mathrm{d}a, \end{aligned}$$

where $\beta (a)$ denotes the disease transmission coefficient at infection age *a*. For model (12), the basic reproduction number ${{\mathcal {R}}}_0$ is given by

$$\begin{aligned} {{\mathcal {R}}}_0 = N \int _0^\infty \beta (a) \mathrm{e}^{-\int _0^a\gamma (\sigma ) \mathrm{d}\sigma } \mathrm{d}a, \end{aligned}$$

where $N=S(t)+\int _0^\infty I(t,a) \mathrm{d}a + R(t)$ denotes the total population. Threshold theorem of ${{\mathcal {R}}}_0$ for an infection age-structured SIR model with vital dynamics was proved by Magal et al. (2010). The concept of the type reproduction number ${{\mathcal {T}}}$ was generalized to the state reproduction number for multi-group infection age-structured models by Inaba and Nishiura (2008).

In contrast, chronological age (time elapsed since the birth) has also been often considered in epidemic models. The SIR model (3) with vital dynamics is generalized to the following system of PDEs:

$$\begin{aligned} \left\{ \begin{array}{ll} \displaystyle \left( \frac{\partial }{\partial t} + \frac{\partial }{\partial a} \right) S(t,a) = -[\lambda (t,a)+\mu (a)] S(t,a), &{} S(t,0)=b, \\ \displaystyle \left( \frac{\partial }{\partial t} + \frac{\partial }{\partial a} \right) I(t,a) = \lambda (t,a) S(t,a) - [\gamma (a)+\mu (a)] I(t,a), \quad &{} I(t,0)=0, \\ \displaystyle \left( \frac{\partial }{\partial t} + \frac{\partial }{\partial a} \right) R(t,a) = \gamma (a) I(t,a) - \mu (a)R(t,a), &{} R(t,0) = 0, \end{array} \right. \end{aligned}$$

where *a* denotes the chronological age and each function is generalized so as to depend on *a*. The force of infection in the mass action law is given by

$$\begin{aligned} \lambda (t,a) = \int _0^\infty \beta (a,\sigma ) I(t,\sigma ) \mathrm{d}\sigma , \end{aligned}$$

where $\beta (a,\sigma )$ denotes the coefficient for disease transmission from infective individuals of age $\sigma$ to susceptible individuals of age *a*. Under appropriate assumptions on each coefficient, the basic reproduction number ${{\mathcal {R}}}_0$ is given by the spectral radius of the following next generation operator (Inaba, 1990):

$$\begin{aligned} {{\mathcal {K}}}\varphi (a) := S^0(a) \int _0^\infty \beta (a,\sigma ) \int _0^\sigma \mathrm{e}^{-\int _\rho ^\sigma [\mu (\eta )+\gamma (\eta )] \mathrm{d}\eta } \varphi (\rho ) \mathrm{d}\rho \mathrm{d}\sigma , \end{aligned}$$

where $\varphi$ is an arbitrary integrable function on ${\mathbb {R}}_+$ and $S^0(a) := b \mathrm{e}^{-\int _0^a \mu (\sigma ) \mathrm{d}\sigma }$ denotes the susceptible population at the disease-free steady state. Although there is no explicit formula of ${{\mathcal {R}}}_0$ in general, we can obtain the following explicit formula in the proportionate mixing case (Dietz & Schenzle, 1985), where $\beta (a,\sigma ) = \beta _1(a)\beta _2(\sigma )$:

$$\begin{aligned} {{\mathcal {R}}}_0 = \int _0^\infty \int _0^\sigma \beta _1(\rho ) \beta _2 (\sigma ) \mathrm{e}^{-\int _\rho ^\sigma [\mu (\eta )+\gamma (\eta )] \mathrm{d}\eta } S^0(\rho ) \mathrm{d}\rho \mathrm{d}\sigma . \end{aligned}$$

Threshold theorem of ${{\mathcal {R}}}_0$ for the chronological age-structured SIR model does not hold in general. In fact, although the global asymptotic stability of the disease-free steady state for ${{\mathcal {R}}}_0 < 1$ was proved by Inaba (1990), the global asymptotic stability of the endemic steady state for ${{\mathcal {R}}}_0 > 1$ does not hold in general (Thieme, 1991). In some cases, periodic solutions exist for ${{\mathcal {R}}}_0 > 1$ (Andreasen, 1995; Franceshetti et al., 2012; Kuniya, 2019).

In application, age-structured epidemic models are often formulated as multi-group ODEs systems. In fact, PDEs models as above can be discretized into such ODEs systems under the assumption that each coefficient is stepwise constant (Tudor, 1985). Therefore, age-structured PDEs models are mathematically general. They enable us to consider the variation of continuous age distributions.

### Seasonality

To take into account the seasonality, model parameters are often assumed to be periodic with respect to time. For instance, we can assume that the disease transmission coefficient $\beta$ and the removal rate $\gamma$ in the SIR model (1) without vital dynamics are periodic with respect to time. That is, for any *t*,

$$\begin{aligned} \beta (t+T)=\beta (t), \quad \gamma (t+T)=\gamma (t), \end{aligned}$$

where $T>0$. The SIR model (1) can then be generalized to the following time-periodic system:

13 $$\begin{aligned} \left\{ \begin{array}{l} \displaystyle S'(t) = -\lambda (t) S(t), \\ \displaystyle I'(t) = \lambda (t) S(t) - \gamma (t) I(t), \\ \displaystyle R'(t) = \gamma (t) I(t), \end{array} \right. \end{aligned}$$

where the force of infection in the mass action law is given by $\lambda (t)=\beta (t)I(t)$. The basic reproduction number ${{\mathcal {R}}}_0$ for model (13) is given by the spectral radius of the following linear operator (Bacaër & Guernaoui, 2006):

$$\begin{aligned} {{\mathcal {K}}} \varphi (t) := N \int _0^\infty \beta (t) \mathrm{e}^{-\int _{t-\tau }^t \gamma (\sigma ) \mathrm{d}\sigma }\varphi (t-\tau ) \mathrm{d}\tau , \end{aligned}$$

where $\varphi$ is an arbitrary *T*-periodic function on ${\mathbb {R}}$. In this case, ${{\mathcal {R}}}_0$ can be explicitly calculated as follows (Bacaër & Guernaoui, 2006, Section 5):

$$\begin{aligned} {{\mathcal {R}}}_0 = \frac{\int _0^T\beta (t)\mathrm{d}t N}{\int _0^T \gamma (t)\mathrm{d}t}. \end{aligned}$$

That is, in this case, ${{\mathcal {R}}}_0$ can be obtained by averaging the periodic parameters. However, in general, there can exist a gap between a quantity obtained by averaging periodic parameters and ${{\mathcal {R}}}_0$ defined by the spectral radius of a linear operator (Bacaër & Ouifki, 2007). ${{\mathcal {R}}}_0$ for more general nonautonomous systems was defined by Inaba (2012, 2019) from the perspective of the generation evolution operator.

As we can easily expect, epidemic models with time-periodic parameters have periodic solutions in many cases (Hethcote & Levin, 1989; Nakata & Kuniya, 2010). The periodicity of such periodic solutions is due to the periodicity (seasonality) of model parameters.

### Diffusion

To consider the spatial spread of infectious diseases, reaction-diffusion systems have been studied (Hosono & Ilyas, 1995). The SIR model (1) without vital dynamics can be generalized to the following SIR model with diffusion:

14 $$\begin{aligned} \left\{ \begin{array}{l} \displaystyle \frac{\partial S(t,x)}{\partial t} = d_1 \varDelta S(t,x) - \lambda (t,x) S(t,x), \\ \displaystyle \frac{\partial I(t,x)}{\partial t} = d_2 \varDelta I(t,x) + \lambda (t,x) S(t,x) - \gamma (x)I(t,x), \\ \displaystyle \frac{\partial R(t,x)}{\partial t} = d_3 \varDelta R(t,x) + \gamma (x)I(t,x), \end{array} \right. \end{aligned}$$

where $x \in \varOmega$ is the space variable and $d_1,d_2$ and $d_3$ are diffusion coefficients for susceptible, infective and removed populations, respectively. Each function is generalized to a function depending on *x*. The force of infection in the mass action law is given by

$$\begin{aligned} \lambda (t,x) = \beta (x) I(t,x). \end{aligned}$$

Reaction–diffusion systems have been used to model the spread of diseases such as rabies, which are transmitted by wild animals (Kallén et al., 1985). The basic reproduction number ${{\mathcal {R}}}_0$ for the diffusive SIR model (14) is given by the spectral radius of the following next generation operator ${{\mathcal {K}}}$:

$$\begin{aligned} {{\mathcal {K}}}\varphi (x) := \beta (x)S^0(x) \int _0^\infty \int _\varOmega \varGamma (t,x,y) \varphi (y) \mathrm{d}y \mathrm{d}t, \end{aligned}$$

where $\varphi$ is an arbitrary continuous function on $\varOmega$, $S^0$ is the susceptible population at the disease-free steady state, and $\varGamma$ is the Green function to the problem

$$\begin{aligned} \frac{\partial u(t,x)}{\partial t} = d_2 \varDelta u(t,x) - \gamma (x) u(t,x) \end{aligned}$$

with appropriate initial and boundary conditions.

For diffusive epidemic models, the threshold principle of ${{\mathcal {R}}}_0$ has been studied in the context of not only the global asymptotic stability of steady states (Allen et al., 2008; Kuniya & Wang, 2017) but also the existence of traveling wave solutions (Hosono & Ilyas, 1995; Adimy et al., 2021). The property of solutions highly depends on the choice of set $\varOmega$ and boundary conditions. For instance, even if model parameters are space-independent, ${{\mathcal {R}}}_0$ could be changed by the shape of the boundary of $\varOmega$ in the case of the Dirichlet boundary conditions (Chekroun & Kuniya, 2020).

### Mutation and reinfection

Effect of the mutation of virus on the epidemic dynamics has also been modeled by the PDEs systems. Pease (1987) proposed an epidemic model to consider the drift and shift of influenza A virus. It can be generalized to the following SIS model:

15 $$\begin{aligned} \left\{ \begin{array}{l} \displaystyle \left( \frac{\partial }{\partial t} + k\frac{\partial }{\partial a} \right) S(t,a) = - \lambda (t,a) S(t,a), \quad kS(t,0) = \gamma I(t), \\ \displaystyle I'(t) = \int _0^\infty \lambda (t,a) S(t,a) \mathrm{d}a - \gamma I(t), \end{array} \right. \end{aligned}$$

where *a* is a variable indicating the immunity level of susceptible individuals. The force of infection in the mass action law is given by $\lambda (t,a) = \beta (a) I(t)$ and $\beta$ is monotone increasing on ${\mathbb {R}}_+$. In this model, as time evolves, the virus mutates and the variable *a* increases with speed *k*, and the susceptible individuals become more susceptible. The basic reproduction number ${{\mathcal {R}}}_0$ for model (15) is given as follows (Inaba, 2017, Section 8.1):

$$\begin{aligned} {{\mathcal {R}}}_0 = \frac{\beta (\infty ) N}{\gamma }, \end{aligned}$$

where $N=\int _0^\infty S(t,a)\mathrm{d}a + I(t)$ is the total population. If ${{\mathcal {R}}}_0 \le 1$, then the disease is eradicated as time evolves, whereas if ${{\mathcal {R}}}_0 > 1$, then there exists a unique endemic steady state (Inaba, 2017, Proposition 8.1). The endemic steady state is not always stable for ${{\mathcal {R}}}_0 > 1$, and periodic solutions can exist in some cases (Magal & Ruan, 2010). The waning of immunity and reinfection have been studied by age structured PDEs (Okuwa et al., 2019) and delay differential equations (Nakata et al., 2014).

## Discussion

In this paper, we have reviewed the structure of basic and advanced epidemic models for the future applications in economics. To construct a suitable model, we suggest to determine

1. compartments to be studied;
2. whether and how the heterogeneity is incorporated into the model;
3. how the effect of intervention policies is taken into account.

For example, as stated in Sect. 2.1, the asymptomatic infection should not be disregarded in the application to COVID-19. Therefore, if we construct a model for COVID-19, then it would be better to include the asymptomatic infective class into the model. Moreover, as the disease-induced death rate of COVID-19 is higher in the elderly people than in the young people, it would be better to incorporate the age structure into the model. If we want to discuss the optimal vaccination policy, then it would be better to consider the age-specific vaccination rate. We may evaluate the effectiveness of the intervention policy by performing the sensitivity analysis of model solutions or reproduction numbers with respect to the vaccination rate.

In this paper, we have reviewed the theory of basic, effective, control and type reproduction numbers. We now summarize the roles of them as follows:

- Basic reproduction number ${{\mathcal {R}}}_0$ represents the essential intensity of epidemic and can determine the model dynamics by the threshold property.
- Effective reproduction number ${{\mathcal {R}}}_t$ represents the real-time intensity of epidemic and can be used to evaluate the effectiveness of interventions.
- Control reproduction number ${{\mathcal {R}}}_\mathrm{c}$ enables us to obtain a critical value for a control parameter to make the reproduction number less than 1.
- Type reproduction number ${{\mathcal {T}}}$ plays a similar role as ${{\mathcal {R}}}_\mathrm{c}$ but it enables us to focus on a control parameter to a specific group in multi-group models.

The theory of epidemic models has developed in decades and accelerated through COVID-19. Nevertheless, it would be still difficult to completely answer to the criticisms raised by Klein et al. (2007). Long-term predictions with constant parameters would contain an essential uncertainty due to the possible change of situation (Kuniya, 2020a). On the other hand, the complexity of models does not necessarily imply the reliability of predictions (Roda et al., 2020). As stated by Huppert and Katriel (2013), the comparison of different results would be important to raise the robustness of predictions. In particular, as there is no single model that can solve all questions (Panovska-Griffths, 2020), we would have to build a scientific attitude to comprehensively understand the results obtained by various researchers from different backgrounds.

In this paper, we have focused only on deterministic models and have not discussed the stochasticity. The stochastic agent-based models have attracted much attention in the period of COVID-19 (Hoertel et al., 2020). They innately consider the interactions between individuals in a heterogeneous population.

In this paper, to model the people’s behavior change, we have mainly focused on the method of nonlinear functions (see Sect. 4.1) and have not discussed other methods such as the utility maximization (Fenichel et al., 2011) and the game theory (Bauch & Earn, 2004), which are rather standard in economics. The author hopes that the mathematical methods reviewed in this paper could contribute to further development of such methods in the context of economic epidemiology.

## Footnotes

1 More rigorous analysis on the existence of periodic solutions has been done in the author’s unpublished manuscript: Kuniya, T. Hopf bifurcation in an SIR epidemic model with psychological effect and distributed time delay, submitted for a chapter in a book entitled *Recent Studies in Mathematical Modeling and Control of the Dynamics of Human Viruses* (Hattaf, K. ed.).
